# Incidence and risk factors of preoperative deep venous thrombosis following pelvic and acetabular fractures: a retrospective case–control study

**DOI:** 10.1186/s13018-022-02972-2

**Published:** 2022-02-05

**Authors:** Weiguang Zhao, Jianlong Zhao, Tiantian Liu, Zhenwu Liu, Li Liu, Yingze Zhang

**Affiliations:** 1 Department of Orthopaedic Surgery, Handan Central Hospital, Handan, 056000 Hebei People’s Republic of China; 2 Hebei North University, Zhangjiakou, 075000 Hebei People’s Republic of China; 3 Chengde Medical University, Chengde, 067000 Hebei People’s Republic of China; 4 Department of Orthopaedic Surgery, Third Hospital of Hebei Medical University, Shijiazhuang, 050051 Hebei People’s Republic of China

## Abstract

The objective of this study was to investigate the prevalence of preoperative deep venous thrombosis (DVT) in the pelvic cavity and lower extremities following pelvic and acetabular fractures and to identify the risk factors of the occurrence of DVT. Duplex ultrasound (DUS) screening and blood tests were conducted in patients admitted from June 2012 to December 2020 for surgical treatment of pelvic and acetabular fractures. Univariate analyses were performed on data of demographics, comorbidities, time from injury to surgery, injury mechanism, accompanied injury, and laboratory results. The optimal cutoff values of continuous variables with statistical significance were obtained by using the receiver operating characteristic (ROC) curve. A multivariate logistic regression analysis was then employed to examine the independent values in terms of predicting preoperative DVT. A total of 607 patients with pelvic and acetabular fractures were included, among whom 82 (13.5%) patients sustained preoperative DVTs. Specifically, 31.7% (26/82) were diagnosed with proximal DVTs. Fifty-two (63.4%) patients had DVT within 7 days after injury, and 67 (81.7%) patients within 10 days. The multivariate logistic regression analysis identified 6 factors independently associated with the presence of preoperative DVT, including age > 46 years (odds ratio [OR] = 2.94), BMI > 26.73 kg/m^2^ (OR = 3.91), time from injury to surgery > 9 days (OR = 5.39), associated injury (OR = 7.85), ALB < 32.8 g/L (OR = 2.71) and FIB > 3.095 g/L (OR = 3.34). Despite the modern prophylactic regimen, the preoperative DVT in patients with pelvic and acetabular fractures still draws the attention of orthopaedic surgeons. Better understanding these risk factors can help surgeons refine the risk stratification profile and perform early interdisciplinary management for patients at high risk of DVT.

## Background

Pelvic and acetabular fractures are commonly caused by blunt high-energy potentially life-threatening injuries, and their perioperative management remains controversial and challenging [1, 2]. Trauma-related endothelial damage, venous stasis, and systematical hypercoagulation pose patients at substantial risk of venous thromboembolic events. DVT sometimes accompanied by pulmonary embolism contributes to the most principal cause of surgical morbidity and mortality in this subpopulation. Without prophylactic treatment, the rate of DVT following pelvic trauma is documented to be high ranging from 35 to 61% with a large proportion of proximal emboli observed [3, 4]. Although extensive regimes for DVT prophylaxis have been explored in orthopaedic patients [5], the rate of the thromboembolic event still varies, from 5 to 34% in this high-risk group [6, 7], which places these patients at increased risk for fatal pulmonary embolism intraoperatively and postoperatively and necessitate aggressive therapy. Therefore, it appears to be particularly significant to highlight early screening of DVT after pelvic trauma.

Consensus on the routine chemoprophylaxis for elective orthopaedic surgeries (hip or knee arthroplasty) has been reached and explicitly validated. However, the importance of thromboprophylaxis following pelvic and acetabular fractures is undervalued due to a lack of refined data of DVT, manifesting large variations in incidence, risk factors, screening methodologies, anatomic locations of clots, and optimal thrombosis [6–9]. DVTs were considered a rare entity in the previous literature, while an increasing body of evidence indicates that Asian patients have a similar prevalence of DVT to that in Caucasians [7, 10, 11]. During the recent decades, Asian countries are bearing a growing incidence of high-velocity traumas, including pelvic and acetabular fractures, which can be treated surgically with satisfactory results [12, 13]. However, there is still no definite information on the characteristics of DVT focusing on the period from pelvic and acetabular fractures to surgical procedures.

Taken together, the data regarding incidence and risk factors specific to the subgroup with pelvic and acetabular fractures are scarce. The objectives of this study included: (1) to investigate the incidence of DVT during the preoperative hospital stay for surgically treated pelvic and acetabular fractures, and (2) to identify the risk factors independently associated with the occurrence of DVT by employing statistical analyses.

## Materials and methods

This retrospective, single-center study was performed following principles of the international guidelines for human research protections, the Declaration of Helsinki, and it was in line with Strengthening the Reporting of Observational Studies in Epidemiology (STROBE) Guidelines. All components of the study were approved by and covered under the Institutional Review Board, Faculty of Medicine, Handan Central Hospital, Handan, China. Informed consent was signed by all the participants. All the patients included in this research were admitted to our hospital from June 2012 to December 2020 for surgical treatment of pelvic and acetabular fractures. The study period started from injury to either occurrence of DVT or operation. All the data were abstracted from the radiology information system and electronic medical record system.

### Inclusion and exclusion criteria

Inclusion criteria were (1) patients admitted to our institution from June 2012 to December 2020 and received surgical treatment, (2) age of 18 years and older, and (3) definite diagnosis of pelvic and/or acetabular fractures with complete medical data. Exclusion criteria were (1) pathological (metastatic) fracture, (2) injury associated with the fracture that required immediate surgical intervention, (3) active malignant tumor, (4) a history of venous thromboembolic disease, or (5) recent use of anticoagulants for other indications within 3 months.

### Data acquisition and variables of interest

The inpatients’ comorbidities and demographic data were retrieved from the electronic medical record system, radiographic image, and operation report system. The information included sex, age, body mass index (BMI), diabetes mellitus, chronic heart disease, hypertension, smoking habits (current smoker or not), alcohol consumption (daily drinker or not). Trauma-related data comprised the following information: (1) type of fracture: pelvic ring fractures being classified according to Young–Burgess classification [14] and acetabular fractures according to Judet–Letournel classification [15]; (2) injury mechanism: lower-energy fracture was defined as a fracture caused by fall from standing height or low height of less than 1 m, and high-energy fracture as from high height, motor vehicle accidents [16]; (3) concomitant injuries (involving the chest, long bone fracture, spine, head, and abdomen); (4) time from injury to the operation of osteosynthesis; and (5) American Society of Anesthesiologists (ASA) classification. Fracture patterns were independently reviewed based on X-rays and computed tomography scans by two senior orthopaedic surgeons to obtain a consistent interpretation of the images. Combined pelvic and acetabular fractures are classified as one certain fracture type according to which was dominant (pelvic or acetabular fracture).

Overnight fasting blood samples were drawn and measured at the central laboratory of our institution according to the manufacturers' instructions. The reference value of each inspection item was determined by the laboratory before reporting the patient's test results. Hematological indicators of interest included albumin (ALB) level, high-density lipoprotein cholesterol (HDL-C) level, low-density lipoprotein cholesterol (LDL-C) level, very-low-density lipoprotein (VLDL) level, red blood cell (RBC) count, hemoglobin (HGB) level, platelet (PLT), prothrombin time (PT), activated partial thromboplastin time (APTT), fibrinogen (FIB) and D-dimer level.

### Diagnosis and prophylaxis of DVT

Venography was taken as the golden standard if DVT could not be ruled out by the duplex ultrasound (DUS) screening. All the patients underwent DUS screening and monitoring on bilateral lower extremities and pelvis with Philips Affiniti50 ultrasonographic machine (Royal Phillips Electronics, Amsterdam, The Netherlands) after admission, every 5–7 days or when any suspected symptoms appeared, according to authors’ institutional protocol. Senior sonographers performed the scanning from the calf veins to iliac veins for positive DVTs. The diagnostic criteria included direct visualization of intraluminal thrombus, the presence of an intraluminal defect, loss of compressibility of the vein, blunted or inadequate flow augmentation, and lack of spontaneous and respirophasic flow above the knee segments [17]. Spiral computed tomography angiography was taken when any symptoms and signs indicative of suspected pulmonary embolism presented or definitive diagnosis of DVT in pelvic veins could not be obtained based on DUS. Proximal DVT was defined if it was localized in popliteal and proximally, and thrombosis that occurred from the distal to popliteal vein was classified as distal DVT. According to ultrasonographic and radiological results, the patients were divided into 2 groups, a DVT group and a non-DVT group.

After admitted to the hospital with a negative result of DVT, all the patients were administered with intermittent pneumatic compression on lower limbs and a prophylactic dose of the low-molecular-weight heparin (enoxaparin, 20–40 mg or dalteparin, 2500–5000 IU, once daily) within 48 h. For those with hemodynamic instability, pharmacological prophylaxis was prescribed once stable. Patients with positive DVTs were given therapeutic anticoagulants (enoxaparin, 20–40 mg or dalteparin, 2500–5000 IU, twice daily), and withheld the mechanical prophylaxis immediately. For patients with proximal DVT, preoperative placement of a retrievable vena cava filter was performed as prophylaxis against intraoperative and postoperative pulmonary embolism. Chemical prophylaxis was stopped 12 h before surgery.

### Statistical analysis

All the statistical analyses were employed by SPSS26.0 (IBM, Armonk, New York, USA). Continuous variables were expressed as mean ± standard deviation (SD)/median (upper quartile, lower quartile). The Kolmogorov–Smirnov test was performed to evaluate the normality of the continuous data, and then, these data were compared between DVT group non-DVT group by using Student-*t* test or Mann–Whitney *U* test, as appropriate. Variables with statistical significance (*p* < 0.05) were analyzed by using the receiver operating characteristic (ROC) curve to obtain the optimal cutoff values associated with the presence of DVT. Afterward, these factors were converted from continuous variables into categorical variables. A Pearson Chi-square test or Fisher's exact test was used, to evaluate the association between inter-groups, expressed as number and percentage. A multivariate logistic regression analysis was performed to examine the independent value of each significant variable from the univariable analyses in terms of predicting the outcome of preoperative DVT. Variables with a p-value less than 0.10 were retained in the final model, and the correlation strength was represented by odds ratio (OR) and 95% confidence interval (95% CI). The fitting degree of the model was evaluated by Hosmer–Lemeshow (H–L) test. Data with a *p*-value less than 0.05 were considered statistically significant.

## Results

### The baseline of the patients

In total, 607 patients with pelvic and acetabular fractures were included in this study. There were 342 males and 265 females with a mean age of 45.4 ± 14.3 years (range:18–90). According to the results of fracture classification, 291 had pelvic fractures (simple type in 159 patients, complex type in 132 patients), and 316 patients sustained acetabular fractures (anterior–posterior compression type in 98 patients, lateral compression type in 189 patients, vertical shear-type in 20 patients, and combined mechanism type in 9) (See Table 1).

**Table 1:** The incidence of DVT in pelvic and acetabular fractures

Classification	No. (%) of patients	No. (%) of DVT
Pelvic fractures	291 (47.9%)	36 (43.9%)
Simple	159 (26.2%)	16 (19.5%)
Complex	132 (21.7%)	20 (24.4%)
Acetabular fractures	316 (52.1%)	47 (57.3%)
APC	98 (16.1%)	11 (13.4%)
LC	189 (31.1%)	19 (23.2%)
VS	20 (3.3%)	12 (14.6%)
CM	9 (1.5%)	5 (6.1%)
Total	607 (100%)	82 (100%)

APC, anterior–posterior compression; LC, lateral compression; VS, vertical shear; CM, combined mechanism

### Characteristics of preoperative DVT

The mean time from injury to the diagnosis of DVT was 6.4 days, ranging from 1 to 16. Of the total 607 patients, 82 patients had DVT before surgery. The overall incidence is 13.5% (82/607) and specifically, 31.7% (26/82) was diagnosed with proximal DVTs, which required placement of a vena cava filter. To be noted, in those with proximal DVTs, only 5 patients had an isolated thrombus in the thigh or pelvic vein, each of the other 21 patients had multiple thrombi in both proximal and distal veins at the first detection time of DVT. There were 13 cases of DVT in bilateral lower limbs and 69 cases in unilateral extremities. Twenty-four cases of DVT occurred in the injured limbs, 3 in uninjured limbs, and 1 in bilateral limbs. The mean time from injury to the diagnosis of DVT was 6.4 days, ranging from 1 to 16. Fifty-two (63.4%) patients had DVT within 7 days after injury, and 67 (81.7%) patients within 10 days. During the research period, no pulmonary embolism or uncontrolled bleeding was observed in patients.

### Optimal cut-off and univariate analysis

The comparison of continuous variables in patients with or without DVT was conducted, and the results are listed in Table 2.

**Table 2:** Comparison of continuous variables in patients with or without preoperative DVT following pelvic and acetabular fracture

Variable	DVT group (*n* = 82) (mean ± SD)	Non-DVT group (*n* = 525) (mean ± SD)	*p* value
Age (years)	51.59 ± 14.8	44.41 ± 13.9	< 0.001*^a^
BMI, kg/m^2^	26.5 ± 3.2	25.00 ± 3.5	0.005*^a^
Time from injury to surgery	12.41 ± 4.1	7.78 ± 4.3	< 0.001*^b^
ALB	32.75 ± 4.8	34.29 ± 5.5	0.017*^a^
HDL-C	0.96 ± 0.3	1.00 ± 0.3	0.311^b^
LDL-C	2.39 ± 0.7	2.32 ± 0.8	0.438^b^
VLDL	0.61 ± 0.3	0.57 ± 0.3	0.292^b^
RBC	3.58 ± 0.5	3.58 ± 0.56	0.993^a^
HGB	110.24 ± 13.8	111.28 ± 17.6	0.813^b^
PLT	305.35 ± 118.1	255.81 ± 107.6	< 0.001*^b^
PT	12.31 ± 1.5	12.37 ± 1.4	0.755^b^
APTT	29.67 ± 3.8	29.34 ± 3.7	0.468^b^
FIB	4.32 ± 1.4	3.74 ± 1.4	0.001*^a^
D-dimer	3.74 ± 7.4	3.19 ± 2.7	0.230^b^

DVT, deep vein thrombosis; BMI, body mass index; ALB, albumin; HDL-C, high-density lipoprotein cholesterol level; LDL-C, low-density lipoprotein cholesterol level; VLDL, very-low-density lipoprotein level; RBC, red blood cell; HGB, hemoglobin; PLT, platelet; PT, prothrombin time; APTT, activated partial thromboplastin time; FIB, fibrinogen

*Statistical significance

^a^Student-*t* test

^b^Mann–Whitney *U* test

**Fig. 1:**
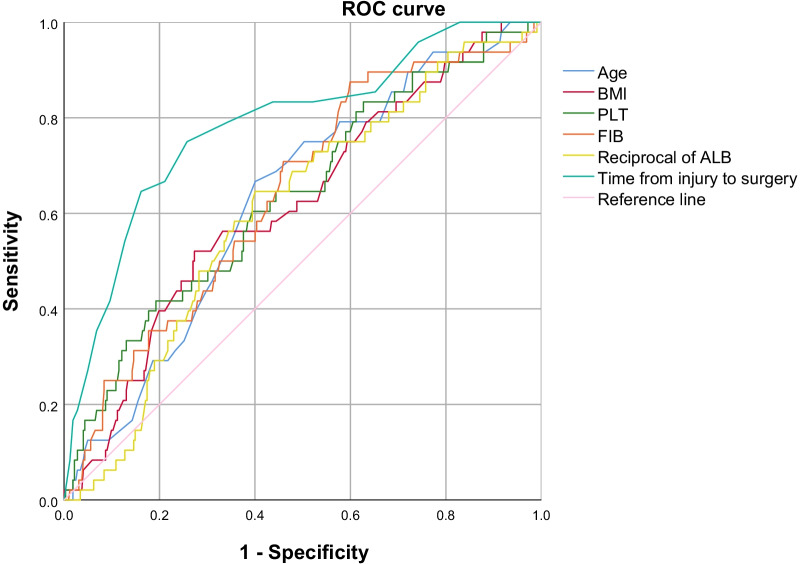
The ROC curve of 6 continuous variables with statistical significance cutoff values. The optimal predictive values of age, BMI, PLT, FIB, ALB and the time from injury to surgery were 46, 26.73, 332.5, 3.09, 32.8 and 9, respectively

As shown in the table, we found significant differences between the two groups concerning age, BMI, the time from injury to surgery, and blood parameters including ALB, PLT, and FIB. Not surprisingly, the ROC analysis showed significant results regarding the above-mentioned factors (see Fig. 1). The optimal cutoff values of the age, BMI, time from injury to surgery, ALB, PLT, and FIB were 46 years, and 26.73 kg/m^2^, 9 days, 32.8 g/L, 332.5 *10^9^/L, and 3.09 g/L, respectively (Table 3). The cutoff value was then used to classify the continuous variable into two categories. The univariate analysis results revealed a statistically significant association between preoperative DVT and factors including age (> 46 years), BMI (> 26.73 kg/m^2^), hypertension, time from injury to surgery (> 9 days), associated injury, ASA classification (III–IV), ALB (< 32.8 g/L), PLT (> 332.5) and FIB (> 3.095 g/L) (see Table 4). Next, the 9 factors were subjected to the multivariate logistic regression analysis.

**Table 3:** The ROC curve analysis of continuous variables with statistical significance

Variable	Cutoff value	Sensitivity (%)	Specificity (%)	AUC (95% CI)	*P* value
Age, years	46	66.7	59.9	0.629 (0.551–0.707)	0.004
BMI (Kg/m^2^)	26.73	52.1	72.7	0.619 (0.536–0.702)	0.008
PLT (*10^9^/L)	332.5	41.7	80.7	0.636 (0.551–0.721)	0.002
FIB (g/L)	3.09	87.5	40.1	0.640 (0.560–0.721)	0.002
ALB (g/L)	32.8	64.6	59.9	0.607 (0.527–0.687)	0.017
Time from injury to surgery, days	9	75.0	74.2	0.786 (0.713–0.858)	< 0.001

**Table 4:** Univariate analyses of categorical variables with interest

Variables	Number (%) of DVT (*n* = 82)	Number (%) of non-DVT (*n* = 525)	*P* value
Gender (male)	54 (65.9)	288 (73.9)	0.128
Age (> 46 years)	55 (67.1)	227 (43.2)	< 0.001*
BMI (> 26.73 kg/m^2^)	42 (51.2)	142 (27.0)	0.001*
Diabetes mellitus	9 (11.0)	89 (17.0)	0.171
Hypertension	20 (24.4)	59 (11.2)	0.001*
Chronic heart disease	8 (9.8)	25 (4.8)	0.064
Current smoking	8 (9.8)	47 (9.0)	0.814
Alcohol consumption	8 (9.8)	47 (9.0)	0.814
Time from injury to surgery (> 9 days)	61 (74.4)	151 (28.8)	< 0.001*
Injury mechanism (high-energy)	72 (87.8)	478 (91.0)	0.349
Accompanied injury	68 (82.9)	132 (25.1)	< 0.001*
ASA classification (III-IV)	47 (57.3)	141 (26.9)	< 0.001*
ALB (< 32.8 g/L)	47 (57.3)	211 (40.2)	0.004*
HDL-C (< 1.1 mmol/L)	57 (69.5)	339 (64.6)	0.382
LDL-C (> 3.37 mmol/L)	4 (4.9)	49 (9.3)	0.184
VLDL (> 0.78 mmol/L)	14 (17.1)	85 (16.2)	0.841
RBC (< lower limit)	71 (86.6)	441 (84.0)	0.549
HGB (< lower limit)	86 (82.9)	425 (81.0)	0.670
PLT (> 332.5)	30 (36.6)	99 (18.9)	< 0.001*
PT < 10 s	1 (1.2)	6 (1.1)	0.952
APTT < 28 s	23 (28.0)	183 (34.9)	0.226
FIB (> 3.095 g/L)	68 (82.9)	302 (57.5)	< 0.001*
D-Dimer (> 0.5 mg/L)	71 (86.6)	410 (78.1)	0.078

ASA, the American Society of Anesthesiologists; RBC, red blood cell, reference range: female, 3.5–5.0*10^12^/L; males, 4.0–5.5*10^12^/L. *HGB* hemoglobin, reference range: females, 110–150 g/L; males, 120–160 g/L

*Statistical significance

### Multivariate logistic regression analysis

The multivariate logistic regression analysis showed 6 factors were identified independently associated with the presence of preoperative DVT. The adjusted results are summarized in Table 5, which were: age > 46 years (OR = 2.94), BMI > 26.73 kg/m^2^ (OR = 3.91), time from injury to surgery > 9 days (OR = 5.39), associated injury (OR = 7.85), ALB < 32.8 g/L (OR = 2.71) and FIB > 3.095 g/L (OR = 3.34). The Hosmer–Lemeshow test demonstrated excellent fitness of the final model (*X*^2^ = 2.472, *p* = 0.963; Nagelkerke *R*^2^ = 0.457).

**Table 5:** Multivariate analysis of risk factors associated with preoperative DVT

Variables	OR	95%CI		*P* value
		Lower limit	Upper limit	
BMI > 26.73 kg/m^2^	3.91	1.787	8.540	0.001
Age > 46 years	2.94	1.387	6.244	0.005
ALB < 32.8 g/L	2.71	1.261	5.830	0.011
FIB > 3.09 g/L	3.34	1.329	8.406	0.010
Associated injury	7.85	3.611	17.056	< 0.001
Time from injury to surgery > 9 days	5.39	2.475	11.735	< 0.001

## Discussion

To the best of our knowledge, this is one of the largest retrospective studies conducted on the incidence and risk factors of preoperative DVT in patients undergoing pelvic and acetabular fractures. Despite the use of the modern prophylactic regimen, our results indicated a 13.5% incidence of DVT before surgery, with 31.7% of those being proximal veins in origin. In addition to some well-established predisposing factors such as older age or obesity, other risk factors also exhibited independent association with preoperative DVT, which was partially consistent with prior studies on population with fractures [18–20]. However, the consensus on DVT management following pelvic and acetabular fractures has not been reached, largely due to their variations in diagnostic method and prophylactic protocol. For example, a prospective study by Kim et al. [7] showed that 33.7% of patients developed DVT screened by indirect CT venography, the higher incidence of which might be due to the absence of chemoprophylaxis. Another study revealed that with the routine use of pharmacological prophylaxis after pelvic trauma, the prevalence of thromboembolic events was 29.1% with more than 60% of those DVTs being proximal [8], while this study did not differentiate the pre-and post-operative DVTs, thus giving rise to the discrepancy between their conclusions and the results of the current study. The previous literature demonstrated that manipulation during surgery predisposed patients with pelvic trauma at higher risk of vascular injury [3]. Hence, we considered the surgery itself another possible risk factor for DVT to focus on the early identification and prevention of DVT following trauma. Overall, our findings showed that patients with pelvic and acetabular fractures had a significantly increased risk of developing DVT and the chemical prevention strategy should be advocated in DVT management during the preoperative hospital stay.

Increased age has been well-acknowledged as one of the principal risk factors for thromboembolic diseases, particularly in trauma patients [7, 20, 21], although no strong association between old age and DVT was found in some literature [9]. In this study, the univariable analysis results showed the mean age of patients with preoperative DVT was 7.18 years older than those without DVT (51.59 years vs 44.41 years) (*p* < 0.001), and the morbidity of DVT in patients aged older than 46 years was 67.1%, significantly higher than the rate of their counterparts (43.2%, *p* < 0.001). After adjusting the confounding factors, we found the patients with pelvic and acetabular fractures over 46 years old had a 2.94-fold increased risk of DVT than that of the younger ones. Similarly, Kim et al. [7] and Wang et al. [8] identified that elderly patients had a greater risk of DVT following pelvic and acetabular fractures. The interrelation between them has not been understood by far, while was considered to relate with increasing presence of other comorbidity or enhanced coagulation potential with aging [22]. Given that the DVT screening strategy and prophylaxis regimen were not age-adjusted in this current study, and the effect of old age on thrombosis was likely to be underestimated in some certain frail elderly patients. Therefore, DVT-related screening and prophylaxis should be highlighted for elderly patients with pelvic ring and acetabular injury.

It has been widely recognized that orthopaedic patients with elevated BMI have a higher risk of perioperative complications than patients within the normal range [23, 24]. However, the impact of elevated BMI on the presence of DVT was not shown in some investigations, partially due to the small sample size as well as to numerous heterogeneities in research objects and study design and screening method [7, 8, 19]. Obesity in the current study was defined as a BMI value over 26.73 kg/m^2^, which was determined according to the ROC analysis. It was found that obesity resulted in 3.91-time increased odds of developing DVT in this trauma group (*p* = 0.001). Similar findings have been reported by Morris et al. [25] who found a significantly increased obesity-related risk of complications including DVT after pelvic and acetabular trauma. In addition, Karunakar et al. [26] reported that patients with BMI ≥ 30 were 2.1-fold more likely to develop DVT than those with BMI < 25 after acetabular fractures. The underlying mechanism was assumed to be related to imbalanced activation of coagulation and inflammation cascades or the large body size that impacted venous return in obese patients [22]. We did not find the correlation between DVT and male sex and medical comorbidities (e.g., diabetes mellitus). It seemed that these factors for DVT were less influential in trauma patients because the high baseline prevalence of DVT tends to overwhelm other effects. Further research on their definitive relationship should be conducted in the future. As an independent risk factor, obesity should be taken into account in predicting the risk of thromboembolic complications when individual risk assessment was conducted for patients following pelvic and acetabular fractures.

In the present study, the incidence of DVT in patients with concurrent injuries was significantly higher than those with isolated pelvic and/or acetabular fractures. The regression model indicated it played an independent role in the formation of a venous clot, although pelvic and acetabular fractures themselves have been generally believed to be at high risk of thromboembolic events. It appeared to be an accumulative effect of polytrauma on amplifying the risk. Our results suggested that patients with accompanied injury demonstrated a remarkably elevated risk of DVT compared to those without other injuries (OR = 7.85, *p* < 0.001), consistent with previously published findings [8, 27]. This was somewhat contrasting with the findings of a prospective study that the rising risk of DVT in patients with pelvic trauma did not correlate with other non-orthopaedic injuries [4]. This discrepancy might be due to that they did not perform serial venography after admission, to some asymptomatic DVT were unable to detect. We did not find evident relation between high-energy injury and the morbidity of DVT. However, we noticed a delay to surgery was intensely associated with elevated incidence of DVT in patients with pelvic and acetabular fractures. To be specific, a 5.39-fold elevated risk of morbidity was encountered among patients who had an interval between injury to surgery > 9 days compared with their counterparts. This finding was compatible with previously published studies. For example, Buerger et al. [28] observed a continuously increased prevalence of DVT as immobilization prolonged after acetabular fracture. Wang et al. [25] revealed the incidence of DVT was significantly increased if the surgical intervention was performed more than 2 weeks after pelvic injury. Another study, however, found that diminished morbidity of DVT was observed in patients who received surgery within 2 days of presentation [29]. This inconsistency could be interpreted that the design of the study obscured the nature of the index procedure and the first surgical intervention might not always be fracture fixation. Based on the consideration of DVT prevention, we favored the view that fixation for pelvic and acetabular fractures was recommended at the earliest opportunity for medically ready patients.

Fibrinogen is a relatively abundant plasma protein, which exerts multiple functions, in fibrin clot formation, platelet aggregation, and inflammation as an acute phase reactant. Our ultimate result suggested that patients with FIB > 3.09 g/L had a 3.34-fold increased risk of DVT. This cutoff value of FIB showed an independent association with thromboembolisms after pelvic and acetabular fractures. It is worth noting that after using multivariate logistic regression analysis, the confounding variable PLT was ruled out, which clarified the association between exposure (FBI) and outcome (DVT) in the certain population. Despite the numerous studies regarding FIB associations, the precise relationship between PLT count and FIB levels has yet to be fully investigated.

Hypoalbuminemia is adversely associated with complications and prognosis after major orthopaedic surgeries [30, 31]. A recent study by Ma et al. [18] found that low albumin level was strongly linked with preoperative DVT in patients undergoing spinal fracture. It was approximately concordant with our findings that patients with a serum albumin less than 32.8 g/L and fibrinogen more than 3.09 g/L had 2.71- and 3.34-time elevated risk of DVT following pelvic and acetabular fractures, respectively. Several investigators reported that hypoalbuminemia-induced low oncotic pressure might lead to a hypercoagulable state, which could be reversed by albumin infusion [32]. It also has indicated that the increased fibrinogen levels could be induced by protein loss, which could, in turn, stimulate the synthesis of fibrinogen in the liver [33]. Although the causal relationship between the two parameters was not elucidated in patients with major trauma, they both exhibited independent associations with the presence of DVT before the fixation of pelvic and acetabular fractures. Therefore, we proposed that for patients with hypoalbuminemia and/or hyperfibrinogenemia, the risk of DVT could be reduced by adjusting therapeutic strategies. Combined with or without other tests, the diagnostic value of plasma D-dimer for venous thrombosis was generally acknowledged in trauma patients. However, there was no significant relation between preoperative DVT and plasma D-dimer at admission. We were cautious in the interpretation of this outcome that early D-dimer level exhibited invalidity in predicting subsequent DVT because only the first test result was included, thus leading to the absence of dynamic results.

We conducted a large investigation on the incidence and risk factors of preoperative DVT in patients undergoing pelvic and acetabular fractures. However, the current study has some limitations. Firstly, this is a single-center, retrospective, case–control study, which carries some inherent weaknesses. For example, the risk factors measured in our study represent associated instead of causal relationships, which should be interpreted with clinical practice. And, some variables that potentially influence the statistical results might not be included in this study. Secondly, the main diagnostic method of DVT is color Doppler ultrasonography that may miss the venous thrombosis in the pelvis, compromising the effectiveness of the analysis results. Thirdly, to improve the internal validity, patients with certain medical conditions (e.g., injury associated with the fracture that required immediate surgical intervention) were excluded, so our findings may be less applicable to patients with such features.

## Conclusion

In summary, the modern prophylactic regimen reduced the incidence of preoperative DVT in patients with pelvic and acetabular fractures to 13.5%, but orthopaedic surgeons’ attention should still be paid to patients with age > 46 years, BMI > 26.73 kg/m^2^, time from injury to surgery > 9 days, associated injury, ALB < 32.8 g/L, and FIB > 3.095 g/L. Better understanding these risk factors can help surgeons refine the risk stratification profile and perform early interdisciplinary management for patients at high risk of DVT.

## Data Availability

All the data will be available upon motivated request to the corresponding author of the present paper.

## Abbreviations

DVT: Deep venous thrombosis
ROC curve: Receiver operating characteristic curve
DUS: Duplex ultrasonography
BMI: Body mass index
ASA: American Society of Anesthesiologists
ALB: Albumin
HDL-C: High-density lipoprotein cholesterol
LDL-C: Low-density lipoprotein cholesterol
VLDL: Very-low-density lipoprotein
RBC: Red blood cell count
HGB: Hemoglobin level
PLT: Platelet level, PTprothrombin time
APTT: Activated partial thromboplastin time
FIB: Fibrinogen
OR: Odds ratio
CI: Confidence interval
SD: Standard deviation
